# Washout CYFRA 21-1: A tool to improve diagnostic accuracy of fine needle aspiration in the diagnosis of metastatic lymph nodes in papillary thyroid cancer

**DOI:** 10.1016/j.heliyon.2024.e31682

**Published:** 2024-05-21

**Authors:** Changwen Huang, Qiangqiang Ge, Qian Wang, Liyuan Ye, Yuejiang Gong

**Affiliations:** a Department of Ultrasound, Shangyu People's Hospital of Shaoxing, Shaoxing, China; b Department of Laboratory Medicine, Shangyu People's Hospital of Shaoxing, Shaoxing, China

**Keywords:** Papillary thyroid cancer, Fine-needle aspiration cytology, Metastasis, Biomarker, CYFRA 21-1, Diagnostic accuracy

## Abstract

Thyroid carcinoma has an increasing incidence of endocrine system cancers. Fine needle aspiration cytology (FNAC) and thyroglobulin (Tg) are the primary diagnostic modalities employed for assessing metastatic lymph nodes (LNs) in thyroid cancer. Due to the limited accuracy, rare patients benefited from these procedures. In this research, we aimed to discover a dependable biomarker that could increase the accuracy of FNAC's ability to diagnose metastatic LNs among patients suffering from papillary thyroid cancer (PTC). From March 2021 to July 2023, 99 LNs from PTC patients who had thyroid ultrasonography suspicions of metastases were examined. All patients underwent FNAC, washout Tg and CYFRA 21-1 measurements. Surgical histology and a subsequent FNAC were utilized to validate the outcomes of LNs. In our study, the optimal cut-off value for CYFRA 21-1 washout fluid was 1.145 ng/mL, with a specificity of 94.00 % (slightly lower than Tg and FNAC at 98 %). However, CYFRA 21-1 demonstrated significantly higher diagnostic sensitivity (85.71 %) and accuracy (86.41 %) compared to Tg (71.43 %, 81.55 %) and FNAC (69.39 %, 80.58 %). Furthermore, FNAC plus washout CYFRA 21-1 performed better in diagnosing the metastatic LNs in PTC than FNAC plus Tg, which may indicate a novel solution for metastatic LNs diagnosis in PTC.

## 1 Introduction

Papillary thyroid cancer (PTC) is the predominant pathological subtype of thyroid cancer, and 30 %–80 % of thyroid cancer patients presenting with cervical lymph node metastasis, leading to adverse patient outcomes, including tumor recurrence or mortality [[1], [2], [3]]. Currently, fine needle aspiration cytology (FNAC) is extensively utilized as a diagnostic tool for PTC to identify lymph node (LN) metastasis. Nevertheless, it is operator-dependent and has a relatively low sensitivity, resulting in a certain rate of false negatives [[4], [5], [6]]. Therefore, there is a pressing need to identify additional biomarkers that could be employed alongside FNAC to enhance the diagnostic accuracy in detecting LN metastasis in PTC.

Thyroglobulin (Tg), a precursor protein for thyroid hormones that are crucial for human growth and metabolic regulation, is considered to have a good diagnostic performance in LN metastasis of thyroid cancers [[6], [7], [8]]. Adding Tg measurement to FNAC can increase the detection rate of PTC-associated LN metastasis by 13 % [9]. However, factors such as thyroidectomy status and immunological assay methods can impact the values for Tg, resulting in current discrepancies in the cut-off values for Tg diagnosis of PTC with metastasis [10,11]. The cytokeratin 19 fragment (CYFRA 21-1) is predominantly found in squamous and simple epithelial cells, and its expression is significantly increased in malignant tumors [12,13]. Elevated CYFRA 21-1 levels demonstrated a significant correlation with the presence of distant metastasis in patients with thyroid cancer [14]. However, the definitive role of CYFRA 21-1 in PTC still need to be explored.

The purpose of this study is to determine CYFRA 21-1's diagnostic significance, which could potentially improve the diagnostic precision of detecting metastatic LN in PTC when used in conjunction with FNAC.

## 2 Materials and methods

### 2.1 Study subjects

Patients (n = 72, >18 years old) with PTC were sequentially enrolled from March 2021 to July 2023 at Shangyu People's Hospital of Shaoxing. The diagnosis of PTC was established according to published criteria [2]. All enrolled patients meet the following conditions: 1) Postoperative histopathological confirmation of primary lesion as PTC, 2) FNAC was performed before surgery, 3) Complete clinical data, including preoperative and postoperative follow-up ultrasound examination, FNAC information, surgical pathology, preoperative washout Tg, and wash out CYFRA 21-1. Exclusion criteria: 1) Postoperative cytologic result is non-PTC; 2) The patient has not undergone neck dissection.

### 2.2 Washout Tg and CYFRA 21-1 measurements

99 LNs from 72 patients were examined in this study. Among these patients, 50 had a single lymph node punctured, 17 had two lymph nodes punctured, and 5 had three lymph nodes punctured. All LNs underwent fine needle aspiration, subsequently undergoing analysis of the aspirated washout for the detection of Tg and CYFRA 21-1 by automatic electrochemical luminescence analyzer (Roche Diagnostics) [15].

### 2.3 US and FNAC of suspicious LNs

The suspicious LNs underwent ultrasound (US)-guided FNAC through an EPIQ 7 sonography system (Philips Medical Systems, Bothell, USA), which was administered by experienced radiologists. LNs were considered suspicious if they exhibited characteristics described in the previous study [16]. Following aspiration, samples were prepared for hematoxylin and eosin (HE) stains as well as Papanicolaou stains.

### 2.4 FNAC and LN assessment

The FNAC was operated by experienced pathologists, and LNs were categorized into two groups according to the postoperative histopathological results: metastatic LN group and benign LN group. Metastatic LN group: FNAC or histopathology were positive. Benign LN group: both histopathology and FNAC were negative.

In this study, the diagnosis of surgical pathology results is considered the golden standard. The sample size calculation formula suggests a required sample size of 48.98 for the metastatic LN group and 34.57 for the benign LN group [17].

### 2.5 Statistical analysis

Student's *t*-test was used to determine statistical significance. The optimal cut-off values were confirmed through the receiver operating characteristic (ROC) curve analysis. P values of 0.05 were considered statistically significant in all analyses conducted with SPSS 22.0 software.

## 3 Results

### 3.1 Overview of study subjects

**Fig. 1:**
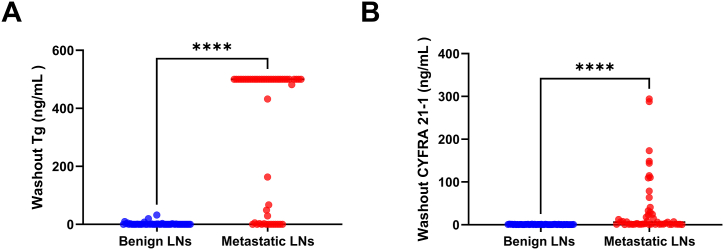
Upregulation of washout Tg and CYFRA 21-1 in patients with metastatic LNs (A). Metastatic LNs had a significantly higher level of washout Tg than that of benign LNs (p < 0.0001, by Mann-Whitney test). (B). Metastatic LNs had a significantly higher level of washout CYFRA 21-1 than that of benign LNs (p < 0.0001, by Mann-Whitney test).

Among 72 patients, 36 patients exhibited no LN metastasis, while the remaining 36 patients had LN metastasis. A total of 8 thyroid tumors smaller than 1 cm were found in the 36 patients with LN metastasis. As indicated in Table 1, regarding age, gender, TSH, and hypertension status, no discernible differences were observed between the two groups. Importantly, the level of Tg was increased in the metastatic group (Fig. 1A). In the metastatic group, CYFRA 21-1 expression was also elevated (Fig. 1B).

**Table 1:** Clinical characteristics of 99 lymph nodes were evaluated in the study.

Clinical Characteristics	Benign LN	Metastatic LN	P value
**Age**			
Mean ± SD	47 ± 11.37	46.9 ± 12.15	0.078
Median	49	44	
Min/Max	25/70	34/84	
**Gender, n (%)**			0.064
Male	7 (19.4 %)	12 (33.3 %)	
Female	29 (80.5 %)	24 (66.7 %)	
**Complication, n (%)**			
Hypertension	6 (16.7 %)	5 (13.9 %)	
**TSH (mU/L)**			
Mean ± SD	1.28 ± 1.15	1.76 ± 1.90	0.137
Median	1.13	1.48	
Min/Max	0.005/4.48	0.033/9.37	
**Washout Tg (ng/mL)**			
Mean ± SD	1.89 ± 5.47	321.12 ± 234.98	<0.0001
Median	0.08	500	
Min/Max	0.04/32.320	0.04/500.00	
**Washout CYFRA 21-1 (ng/mL)**			
Mean ± SD	0.74 ± 0.28	36.82 ± 69.11	<0.0001
Median	0.735	6.13	
Min/Max	0.210/1.450	0.427/294.100	

Abbreviations: TSH, thyroid stimulating hormone; Tg, thyroglobulin; CYFRA 21-1, cytokeratin 19 fragment; SD, standard deviation; Min/Max, minimum/maximum.

### 3.2 Tg and CYFRA 21-1 cut-off values

**Fig. 2:**
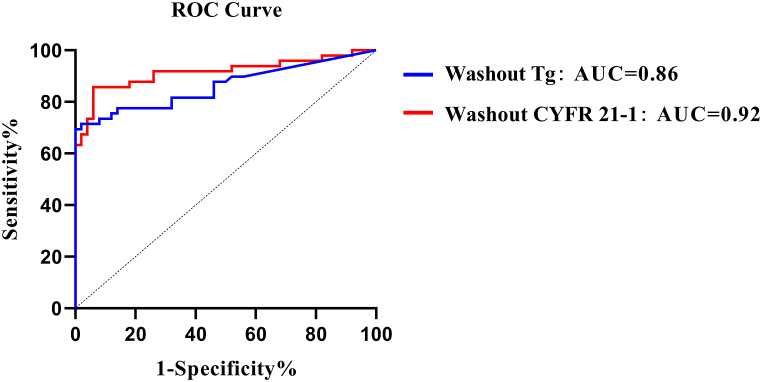
ROC analysis of washout Tg (blue) and washout CYFRA 21-1 (red) tested in lymph node fine-needle aspiration. Metastases were determined using a cut-off value of 24.555 ng/mL (AUC = 0.86, sensitivity = 71.43 %, specificity = 98.00 %) for washout Tg and 1.145 ng/mL (AUC = 0.92, sensitivity = 85.71 %, specificity = 94.00 %) for washout CYFRA 21-1, respectively. (For interpretation of the references to colour in this figure legend, the reader is referred to the Web version of this article.)

As for washout Tg, it shows an AUC of 0.86, sensitivity of 71.43 %, and specificity of 98.0 %, whereas for washout CYFRA 21-1, it shows an AUC of 0.92, sensitivity of 85.71 %, and specificity of 94.00 % (Fig. 2).

### 3.3 Diagnostic performance of FNAC, Tg and CYFRA 21-1

According to surgical pathology results, the diagnostic performance has been summarized in Table 2. Among 49 metastatic LNs, FNAC missed 15, whereas washout Tg and washout CYFRA 21-1 failed to detect 14 and 7, respectively. Within the 50 benign LNs, both FNAC and washout Tg erroneously classified 1 LN as metastatic, whereas washout CYFRA 21-1 misclassified 3 LNs.

**Table 2:** Individual diagnostic performance of FNAC, Washout Tg, and Washout CYFRA 21-1, and their combination according to the outcomes.

	TP	TN	FN	FP
FNAC	34	49	15	1
Washout Tg	35	49	14	1
Washout CYFRA 21-1	42	47	7	3
FNAC + Washout Tg	38	48	11	2
FNAC + Washout CYFRA 21-1	42	46	7	4
FNAC + Washout CYFRA 21-1+Washout Tg	44	45	5	5

Abbreviations: TP, true positive; TN, true negative; FN, false negative; FP, false positive; FNAC, fine needle aspiration cytology; Tg, thyroglobulin; CYFRA 21-1, cytokeratin 19 fragment.

### 3.4 FNAC, Tg, CYFRA 21-1 and their combined diagnostic values

According to the above results, the diagnostic values were evaluated (Table 3). There was a higher specificity for FNAC and Tg than CYFRA 21-1 (98.00 %, 98.00 %, and 94.00 %, respectively), but lower sensitivity (69.39 %, 71.43 %, 85.71 %, respectively). CYFRA 21-1 exhibited a higher negative predictive value (NPV, 87.04 %, 76.56 %, and 77.78 %, respectively) and a diagnostic accuracy (86.41 %, 80.58 %, and 81.55 %, respectively) than FNAC and Tg, suggesting the combination of these items may improve diagnostic accuracy. FNAC plus Tg and FNAC plus CYFRA 21-1 both resulted in an improved the sensitivity (77.55 % and 85.71 %) and NPV (81.36 % and 86.79 %) as compared to FNAC alone. Notably, FNAC plus CYFRA 21-1 demonstrated superior performance in enhancing sensitivity (85.71 % vs 77.55 %), NPV (86.79 % vs 81.36 %), and diagnostic accuracy (85.44 % vs 83.50 %). Compared to FNAC plus Tg, it has a slight disadvantage in specificity (92.00 % vs 96.00 %) and positive predictive value (PPV, 91.30 % vs 95.00 %). FNAC and Tg were combined with CYFRA 21-1 to achieve the greatest sensitivity (89.80 %) and NPV (90.00 %), but the overall diagnostic accuracy (86.41 %) has not been significantly improved.

**Table 3:** Comparison of diagnostic values of FNAC, washout Tg, washout CYFRA 21-1 and their combinations.

Diagnostic Tool	Diagnostic Value				
	Sensitivity (%)	Specificity (%)	PPV (%)	NPV (%)	Accuracy (%)
FNAC	69.39	98.00	97.14	76.56	80.58
Washout Tg	71.43	98.00	97.22	77.78	81.55
Washout CYFRA 21-1	85.71	94.00	93.33	87.04	86.41
FNAC + Washout Tg	77.55	96.00	95.00	81.36	83.50
FNAC + Washout CYFRA 21-1	85.71	92.00	91.30	86.79	85.44
FNAC + Washout CYFRA 21-1 + Washout Tg	89.80	90.00	89.80	90.00	86.41

Abbreviations: PPV, positive predictive value; NPV, negative predictive value; FNAC, fine needle aspiration cytology; Tg, thyroglobulin; CYFRA 21-1, cytokeratin 19 fragment.

## 4 Discussion

Timely diagnosis and standardized treatment can achieve a high disease-free survival (DFS) rate for the majority of PTC patients [18]. Nowadays, ultrasound-guided FNAC has gained widespread acceptance in thyroid surgery [19]. However, the accuracy of FNAC was not ideal in some thyroid tumors, and histology was often needed for diagnosis [5]. Additionally, the limitations of FNA detection also manifested in the diagnosis of larger thyroid nodules (diameter >3 cm) [20]. Thus, if combined with molecular diagnostic methods, the detection range of FNA in thyroid nodules can be improved.

In thyroid cancer, FNA Tg assessment was introduced in 1992 and has demonstrated its superiority in sensitivity compared to FNAC. Its integration with FNAC enhances diagnostic precision and sensitivity [21,22]. Although diagnostic sensitivity is increased, the diagnostic threshold remains undetermined, and interference from elevated serum anti-thyroglobulin antibodies (TgAb) levels may lead to false-negative outcomes [10]. Cytokeratin 19 (CK19) is part of the epithelial cytoskeleton, with tissue overexpression in differentiated thyroid carcinoma (DTC), particularly in cases of papillary carcinoma [23]. Soluble CK19 fragments, known as CYFRA 21-1, released by cancer cells can serve as diagnostic or prognostic markers for lung, intrahepatic cholangiocarcinoma, and breast cancers [[24], [25], [26]]. According to a previous study, thyroid tumors with distant metastases may have higher CYFRA 21-1 levels than thyroid malignancies without metastases [14]. In patients with DTC, CYFRA 21-1 can serve as an indicator for prognostic assessment [27]. In our study, metastatic LN patients had elevated levels of washout Tg and CYFRA 21-1, aligning with previous findings. Furthermore, combining FNAC and CYFRA 21-1 exhibited improved diagnostic performance compared to FNAC alone in detecting metastatic pleural fluids from adenocarcinomas [28]. Moreover, our study found that washout fluid with CYFRA 21-1 at 1.145 ng/mL had the best cutoff value, exhibiting a specificity of 94.00 %, slightly lower than the 98 % specificity of Tg. However, its diagnostic sensitivity was 85.71 %, significantly surpassing the 71.43 % sensitivity of Tg. Besides, combining CYFRA 21-1 with FNAC showed enhanced sensitivity, diagnostic accuracy and NPV compared to the other approaches, such as FNAC, Tg, and FNAC plus Tg assessment. Notably, the combination of CYFRA 21-1, FNAC, and Tg achieved the highest sensitivity (89.80 %) and NPV of 90.00 % compared to other evaluated methods, indicating that CYFRA 21-1 could potentially improve FNAC's diagnostic performance for LN metastasis prediction.

There are some limitations to our study, such as its relatively small sample size, which necessitates further investigation on a larger cohort to validate our findings. Additionally, we lack long-term follow-up data on patients which could offer insights into the accuracy of molecular diagnostics and the efficacy of treatments.

## Declarations

Ethics statement: The study has obtained human research ethics approval from the Ethics Committee of Shangyu People's Hospital of Shaoxing, January 1st, 2021, Approval NO. SRY-20210101-0001.

## Funding statement

This work was supported by the Shaoxing Basic Public Welfare Project (2022A14037).

## Data availability statement

The data that support the findings of this study are available from the corresponding authors upon request.

## CRediT authorship contribution statement

**Changwen Huang:** Writing – review & editing, Methodology, Investigation, Formal analysis, Data curation, Conceptualization. **Qiangqiang Ge:** Writing – review & editing, Validation, Methodology, Investigation, Formal analysis, Data curation. **Qian Wang:** Writing – original draft, Validation, Formal analysis. **Liyuan Ye:** Writing – original draft, Validation, Formal analysis. **Yuejiang Gong:** Writing – review & editing, Supervision, Conceptualization.

## Declaration of competing interest

The authors declare the following financial interests/personal relationships which may be considered as potential competing interests:Yuejiang Gong reports financial support was provided by Shaoxing Science Technology Bureau. If there are other authors, they declare that they have no known competing financial interests or personal relationships that could have appeared to influence the work reported in this paper.

## References

[1] Cabanillas M.E., McFadden D.G., Durante C. (2016). Thyroid cancer. Lancet..

[2] Haugen B.R., Alexander E.K., Bible K.C., Doherty G.M., Mandel S.J., Nikiforov Y.E. (2015). American thyroid association management guidelines for adult patients with thyroid nodules and differentiated thyroid cancer: the American thyroid association guidelines task force on thyroid nodules and differentiated thyroid cancer. Thyroid.

[3] Leite V. (2018). The importance of the 2015 American thyroid association guidelines for adults with thyroid nodules and differentiated thyroid cancer in minimising overdiagnosis and overtreatment of thyroid carcinoma. Eur. Endocrinol..

[4] Domanski H.A. (2020). Role of fine needle aspiration cytology in the diagnosis of soft tissue tumours. Cytopathology : official journal of the British Society for Clinical Cytology..

[5] Feldkamp J., Fuhrer D., Luster M., Musholt T.J., Spitzweg C., Schott M. (2016). Fine needle aspiration in the investigation of thyroid nodules. Dtsch Arztebl Int.

[6] Koshino S., Hamaya H., Ishii M., Kojima T., Urano T., Yamaguchi Y. (2016). Efficacy of fine-needle aspiration cytology in the diagnosis of primary thyroid lymphoma for elderly adults. J. Am. Geriatr. Soc..

[7] Coscia F., Taler-Verčič A., Chang V.T., Sinn L., O'Reilly F.J., Izoré T. (2020). The structure of human thyroglobulin. Nature.

[8] Carpi A., Di Coscio G., Iervasi G., Antonelli A., Mechanick J., Sciacchitano S. (2008). Thyroid fine needle aspiration: how to improve clinicians' confidence and performance with the technique. Cancer Lett..

[9] Al-Hilli Z., Strajina V., McKenzie T.J., Thompson G.B., Farley D.R., Regina Castro M. (2017). Thyroglobulin measurement in fine-needle aspiration improves the diagnosis of cervical lymph node metastases in papillary thyroid carcinoma. Ann. Surg Oncol..

[10] Jo K., Kim M.H., Lim Y., Jung S.L., Bae J.S., Jung C.K. (2015). Lowered cutoff of lymph node fine-needle aspiration thyroglobulin in thyroid cancer patients with serum anti-thyroglobulin antibody. Eur. J. Endocrinol..

[11] Jeon M.J., Park J.W., Han J.M., Yim J.H., Song D.E., Gong G. (2013). Serum antithyroglobulin antibodies interfere with thyroglobulin detection in fine-needle aspirates of metastatic neck nodes in papillary thyroid carcinoma. J. Clin. Endocrinol. Metab..

[12] Pujol J.L., Grenier J., Daurès J.P., Daver A., Pujol H., Michel F.B. (1993). Serum fragment of cytokeratin subunit 19 measured by CYFRA 21-1 immunoradiometric assay as a marker of lung cancer. Cancer Res..

[13] Qiao Y., Chen C., Yue J., Yu Z. (2019). Tumor marker index based on preoperative SCC and CYFRA 21-1 is a significant prognostic factor for patients with resectable esophageal squamous cell carcinoma. Cancer Biomarkers.

[14] Jeong C., Lee J., Yoon H., Ha J., Kim M.H., Bae J.S. (2021). Serum CYFRA 21.1 level predicts disease course in thyroid cancer with distant metastasis. Cancers.

[15] Kristjansdottir B., Partheen K., Fung E.T., Marcickiewicz J., Yip C., Brännström M. (2012). Ovarian cyst fluid is a rich proteome resource for detection of new tumor biomarkers. Clin. Proteonomics.

[16] Lee J., Park H.L., Jeong C.-W., Ha J., Jo K., Kim M.-H. (2019). CYFRA 21-1 in lymph node fine needle aspiration washout improves diagnostic accuracy for metastatic lymph nodes of differentiated thyroid cancer. Cancers.

[17] Hajian-Tilaki K. (2014). Sample size estimation in diagnostic test studies of biomedical informatics. J. Biomed. Inf..

[18] Vasileiadis I., Boutzios G., Karalaki M., Misiakos E., Karatzas T. (2018). Papillary thyroid carcinoma of the isthmus: total thyroidectomy or isthmusectomy?. Am. J. Surg..

[19] Shimura H., Matsumoto Y., Murakami T., Fukunari N., Kitaoka M., Suzuki S. (2021). Diagnostic strategies for thyroid nodules based on ultrasonographic findings in Japan. Cancers.

[20] Shin J.J., Caragacianu D., Randolph G.W. (2015). Impact of thyroid nodule size on prevalence and post-test probability of malignancy: a systematic review. Laryngoscope.

[21] Pacini F., Fugazzola L., Lippi F., Ceccarelli C., Centoni R., Miccoli P. (1992). Detection of thyroglobulin in fine needle aspirates of nonthyroidal neck masses: a clue to the diagnosis of metastatic differentiated thyroid cancer. J. Clin. Endocrinol. Metab..

[22] Cunha N., Rodrigues F., Curado F., Ilhéu O., Cruz C., Naidenov P. (2007). Thyroglobulin detection in fine-needle aspirates of cervical lymph nodes: a technique for the diagnosis of metastatic differentiated thyroid cancer. Eur. J. Endocrinol..

[23] Cameron B.R., Berean K.W. (2003). Cytokeratin subtypes in thyroid tumours: immunohistochemical study with emphasis on the follicular variant of papillary carcinoma. J. Otolaryngol..

[24] Ren H., Hu Y., Xie T., Jin C., Hu Y., Yang B. (2019). Effect of gefitinib on serum EGFR and CYFRA21-1 in patients with advanced non-small cell lung cancer. Oncol. Lett..

[25] Guowei H., Yuan L., Ma L., Zhongyang L., Zhixing S., Lin L. (2019). The diagnostic efficacy of CYFRA21-1 on intrahepatic cholangiocarcinoma: a meta-analysis. Clin Res Hepatol Gastroenterol.

[26] Won S.Y., Kim E.K., Moon H.J., Yoon J.H., Park V.Y., Kim M.J. (2020). [Diagnostic value of CYFRA 21-1 measurement in fine-needle aspiration washouts for detection of axillary recurrence in postoperative breast cancer patients]. Taehan Yongsang Uihakhoe Chi..

[27] Giovanella L., Imperiali M., Trimboli P. (2017). Role of serum cytokeratin 19 fragment (Cyfra 21.1) as a prognostic biomarker in patients with differentiated thyroid cancer. Sci. Rep..

[28] Barillo J.L., da Silva Junior C.T., Silva P.S., de Souza J.B.S., Kanaan S., Xavier A.R. (2018). Increased cytokeratin 19 fragment levels are positively correlated with adenosine deaminase activity in malignant pleural effusions from adenocarcinomas. Dis. Markers.

